# Selection of Antibody Responses Associated With *Plasmodium falciparum* Infections in the Context of Malaria Elimination

**DOI:** 10.3389/fimmu.2020.00928

**Published:** 2020-05-15

**Authors:** Lotus L. van den Hoogen, Gillian Stresman, Jacquelin Présumé, Ithamare Romilus, Gina Mondélus, Tamara Elismé, Alexandre Existe, Karen E. S. Hamre, Ruth A. Ashton, Thomas Druetz, Vena Joseph, James G. Beeson, Susheel K. Singh, Jacques Boncy, Thomas P. Eisele, Michelle A. Chang, Jean F. Lemoine, Kevin K. A. Tetteh, Eric Rogier, Chris Drakeley

**Affiliations:** ^1^Department of Infection Biology, London School of Hygiene & Tropical Medicine, London, United Kingdom; ^2^Center for Applied Malaria Research and Evaluation, Tulane University School of Public Health & Tropical Medicine, New Orleans, LA, United States; ^3^Laboratoire National de Santé Publique, Port-au-Prince, Haiti; ^4^Malaria Branch, Division of Parasitic Diseases and Malaria, Centers for Disease Control and Prevention, Atlanta, GA, United States; ^5^CDC Foundation, Atlanta, GA, United States; ^6^Department of Social and Preventive Medicine, University of Montreal School of Public Health, Montreal, QC, Canada; ^7^Burnet Institute, Melbourne, VIC, Australia; ^8^Department of Medicine, The University of Melbourne, Melbourne, VIC, Australia; ^9^Central Clinical School and Department of Microbiology, Monash University, Clayton, VIC, Australia; ^10^Department of Congenital Disorders, Statens Serum Institut, Copenhagen, Denmark; ^11^Department of Immunology and Microbiology, Centre for Medical Parasitology, University of Copenhagen, Copenhagen, Denmark; ^12^Ministère de la Santé Publique et de la Population, Port-au-Prince, Haiti

**Keywords:** malaria, immunoglobulin G (IgG), multiplex bead assay, sero-surveillance, elimination, ETRAMP

## Abstract

In our aim to eliminate malaria, more sensitive tools to detect residual transmission are quickly becoming essential. Antimalarial antibody responses persist in the blood after a malaria infection and provide a wider window to detect exposure to infection compared to parasite detection metrics. Here, we aimed to select antibody responses associated with recent and cumulative exposure to malaria using cross-sectional survey data from Haiti, an elimination setting. Using a multiplex bead assay, we generated data for antibody responses (immunoglobulin G) to 23 *Plasmodium falciparum* targets in 29,481 participants across three surveys. This included one community-based survey in which participants were enrolled during household visits and two sentinel group surveys in which participants were enrolled at schools and health facilities. First, we correlated continuous antibody responses with age (Spearman) to determine which showed strong age-related associations indicating accumulation over time with limited loss. AMA-1 and MSP-1_19_ antibody levels showed the strongest correlation with age (0.47 and 0.43, *p* < 0.001) in the community-based survey, which was most representative of the underlying age structure of the population, thus seropositivity to either of these antibodies was considered representative of cumulative exposure to malaria. Next, in the absence of a gold standard for recent exposure, we included antibody responses to the remaining targets to predict highly sensitive rapid diagnostic test (hsRDT) status using receiver operating characteristic curves. For this, only data from the survey with the highest hsRDT prevalence was used (7.2%; 348/4,849). The performance of the top two antigens in the training dataset (two-thirds of the dataset; *n* = 3,204)—Etramp 5 ag 1 and GLURP-R0 (area-under-the-curve, AUC, 0.892 and 0.825, respectively)—was confirmed in the test dataset (remaining one-third of the dataset; *n* = 1,652, AUC 0.903 and 0.848, respectively). As no further improvement was seen by combining seropositivity to GLURP-R0 and Etramp 5 ag 1 (*p* = 0.266), seropositivity to Etramp 5 ag 1 alone was selected as representative of current or recent exposure to malaria. The validation of antibody responses associated with these exposure histories simplifies analyses and interpretation of antibody data and facilitates the application of results to evaluate programs.

**Graphical Abstract:**
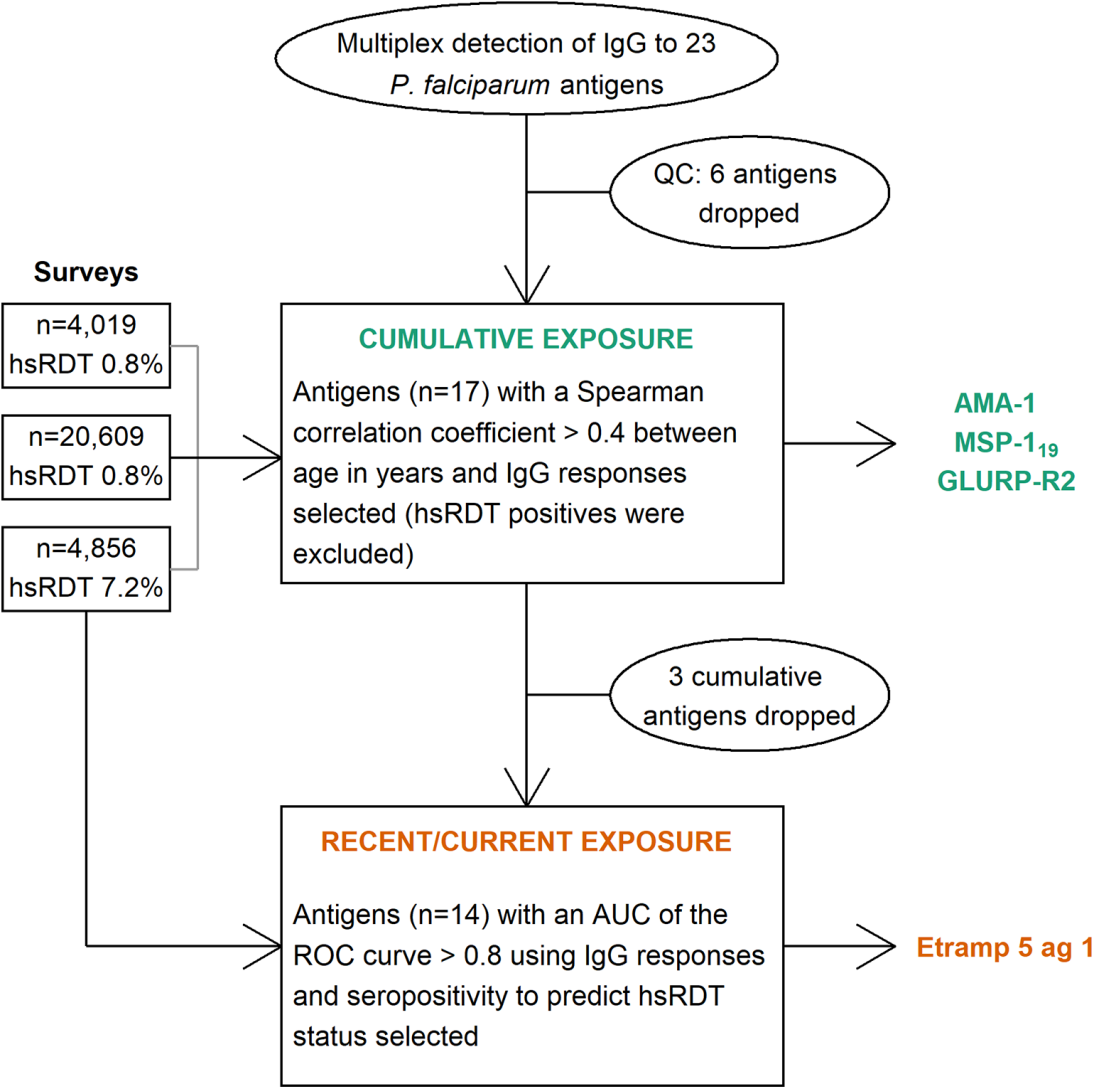
Flowchart of the selection process for immunoglobulin G (IgG) responses to Plasmodium falciparum antigens representative of cumulative and current or recent exposure. QC: quality control, hsRDT: highly sensitive rapid diagnostic test, AUC: area under the curve, ROC: receiver operating characteristic, AMA-1: apical membrane antigen 1, MSP-119: 19 kilodalton fragment of merozoite surface protein 1, GLURP-R2: glutamate rich protein R2, Etramp 5 ag 1: early transcribed membrane protein 5.

## Introduction

Globally, the number of countries in malaria pre-elimination and elimination phases is increasing (1). For malaria control and elimination purposes, the allocation of national resources is commonly guided by regional case counts. However, trends in passively detected cases are dependent on health system coverage and treatment-seeking behavior, and thus may not represent the true malaria burden in all settings. Serological endpoints, typically the presence of antibody responses in resident populations, represent a population's exposure history and can identify areas with residual malaria transmission more accurately than the prevalence of infection at low transmission (2).

Until recently, sero-surveillance has primarily focused on antibodies representing cumulative exposure, such as *Plasmodium falciparum* apical membrane antigen 1 (AMA-1) and the 19 kDa fragment of merozoite surface protein 1 (MSP-1_19_) (2). Age-specific increases in seroprevalence to these antigens, estimated as seroconversion rates (SCR), have been shown to be strongly correlated with entomological inoculation rates (EIR), the gold standard metric for transmission intensity, and with parasite prevalence (2). Antibodies to these antigens persist in the blood with repeated exposure. For MSP-1_19_, model estimates suggested the time to sero-reversion is 23 (3) to 50 years or more (4), while limited data from observational studies suggest half-lives of long-lived antibody secreting cells to be 2 (5) to 16 (6) years. Although MSP-1_19_ and AMA-1 antibody half-lives might be faster in children (7, 8), this may be due to insufficient repeated exposure in children at low transmission. Antibodies with shorter half-lives [i.e., those indicating incidence in the past year (9)] may be able to detect if and where changes in malaria transmission intensity take place more rapidly and accurately as compared to antibodies with long half-lives. Several potential candidates have recently been optimized for use in multiplex bead assays (MBA) (10).

As part of Haiti's aim to eliminate malaria (11), large-scale cross-sectional surveys were performed to assess if and where residual transmission, potentially undetected via routine surveillance, is occurring. Here, we assessed antibody responses to 23 *P. falciparum* recombinant proteins and peptides in 29,481 participants residing in two areas with different levels of transmission intensity. Our aim was to select antibodies associated with cumulative and current or recent exposure to malaria for the Haitian context in order to simplify analyses and interpretation of collected survey data that can be used to inform program decisions.

## Methods

### Study Population

The island of Hispaniola, consisting of Haiti and the Dominican Republic, is the last remaining region in the Caribbean with malaria transmission. In 2016, 97% of the reported malaria cases on the island occurred in Haiti (12). In Haiti, transmission is highly focal as the Grand'Anse department, in the southwestern part of the country, accounted for 47% of the national malaria cases reported in 2017 (13). Three large-scale cross-sectional surveys were conducted in Haiti in 2017 (14): (1) in Artibonite, central Haiti, a community-based household survey (HH-Artibonite) with 21,891 participants; (2) an easy-access-group (EAG) survey in the same area (EAG-Artibonite) including 6,006 participants; and (3) an EAG survey was performed with the same protocol in Grand'Anse, southwestern Haiti, (EAG-Grand'Anse) with 5,034 participants (Table 1). For the EAG surveys, participants were enrolled at schools and health facilities. Although in EAG-Artibonite participants were also sampled at church venues, these were excluded from further analyses due to practical and logistical restraints of this sentinel group (16). At health facilities, participants were enrolled irrespective of the reason for their visit, and both treatment-seeking patients as well as accompanying people were asked to participate. In the HH-Artibonite survey, households were randomly selected in a defined geographic area and all household members were asked to participate; participants were tested at their homes (Hamre et al., in preparation). In both survey types, people were asked to participate irrespective of the presence of fever or symptoms of malaria. Participants from all three cross-sectional surveys provided finger-prick blood for malaria diagnosis by a conventional rapid diagnostic test (cRDT; SD Bioline Malaria Antigen P.f., 05FK50, Standard Diagnostics) and a highly-sensitive RDT (hsRDT; also known as ultrasensitive RDT, Alere Malaria Ag P.f., 05FK141, Standard Diagnostics). In addition, finger-prick blood was spotted on Whatman 903 cards (GE Healthcare), dried overnight at ambient temperature and packed the next day with silica gel. Dried blood spots (DBS) were stored at +4°C and transported to the national laboratory (*Laboratoire National de Santé Publique*; LNSP) in Port-au-Prince weekly. Demographic and household characteristics were collected through a tablet-based questionnaire. Participants testing positive by cRDT were treated according to national guidelines.

**Table 1:** General characteristics of the study population for three malaria cross-sectional surveys performed in Haiti.

**Characteristic**	**HH-Artibonite**	**EAG-Artibonite**	**EAG-Grand'Anse**
Department	Artibonite, central Haiti	Artibonite, central Haiti	Grand'Anse, southwestern Haiti
Communes	Verrettes & La Chappelle	Verrettes & La Chappelle	Anse-d'Hainault, Chambellan, Dame-Marie, Les Irois & Moron
Timing	Jul-Oct 2017^*^	May-Jun 2017	Nov-Dec 2017
Survey type	Community-based household survey	Easy-access-group survey: participants recruited in churches, schools & health facilities	Easy-access-group survey: participants recruited in schools & health facilities
N			
- Available IgG data^**^	21,235	5,898	4,967
- Merged to field data	21,214	4,154^***^	4,959
- Aged ≥1 year	20,609	4,019	4,856
Median age, interquartile range	20, 8–41	12, 8–24	13, 8–22
Highly sensitive RDT prevalence, n/N	0.76%, 157/20,556	0.77%, 31/4,019	7.18%, 348/4,849

*RDT: rapid diagnostic test. IgG: immunoglobulin G*.

* *Two-week pause due to hurricanes*.

** *For more information on the number samples for which IgG data was successfully collected see van den Hoogen et al. (15)*.

*** *The large decrease in samples is due to the exclusion of participants recruited at church venues from further analyses due to practical and logistical restraints of this sentinel group (16)*.

### Antigen Panel and Covalent Coupling of Antigens to Beads

Twenty-three *P. falciparum* antigens and peptides from asexual life-cycle stages were included in the panel (Table 2). Antigens were covalently coupled to unique bead regions as described by Rogier et al. (34). Details on antigen to bead coupling conditions are described elsewhere for most of the antigens in the panel (15), while for the remaining six antigens these are provided in Supplementary Table 1.

**Table 2:** Characteristics of multiplex bead assay *Plasmodium falciparum* antigen panel.

**Antigen**	**Antigen acronym**	**Alias**	**Life cycle stage/location**	**Expression tag**	**Strain**	**Reference**
Circumsporozoite surface protein	rCSP	rcsp	Sporozoite	N/A	3D7	(17)
Liver surface antigen 1	LSA-1	lsa1	Infected hepatocyte	N/A	Synthesized peptide, Pl1043 epitope	(18)
Plasmodium exported protein	Hyp 2	hyp2	Hypothesised location: iRBC	GST	3D7	(9); K.K.A. Tetteh, unpublished
Heat shock protein 40	HSP40 ag 1	hsp40	iRBC	GST	3D8	(9); K.K.A. Tetteh, unpublished
Schizont egress antigen	SEA-1	sea	iRBC	GST	3D7	(19); K.K.A. Tetteh, unpublished
Skeleton-binding protein; Maurer's cleft	SBP1	sbp1	iRBC	GST	3D7	(20); K.K.A. Tetteh, unpublished
Histidine rich protein 2	HRP2	hrp2	iRBC and secreted	GST	Type A and B	(21)
Early transcribed membrane protein	Etramp 4 ag 2	etr4	iRBC, PVM	GST	3D7	(9); K.K.A. Tetteh, unpublished
Early transcribed membrane protein	Etramp 5 ag 1	etr5	iRBC, PVM	GST	3D7	(22); K.K.A. Tetteh, unpublished
Gametocyte exported protein 18	GEXP18	gexp	iRBC/ Gametocyte	GST	3D7	(9); K.K.A. Tetteh, unpublished
CH150/9 allele of MSP2; full-length.	MSP2 CH150/9	msp2_ch150	Merozoite surface	GST	CH150/9	(23)
Dd2 allele of MSP2; full-length.	MSP2 Dd2	msp2_dd2	Merozoite surface	GST	Dd2	(24)
Glutamate rich protein R0	GLURP R0	glurp0	Merozoite surface	N/A	Synthesized peptide, R0 fragment	(25)
Glutamate rich protein R2	GLURP R2	glurp2	Merozoite surface	His_x6_	F32	(26)
19kDa fragment of MSP1 molecule	PfMSP-1_19_	msp119	Merozoite surface	GST	Wellcome	(27)
H103/merozoite surface protein 11	H103/MSP11	h103	Merozoite surface/rophtry neck	GST	3D7	(28)
Erythrocyte binding antigen-140 Region III-V	EBA-140 RIII-V	e140	Merozoite (Micronemes)	GST	3D7	(29)
Erythrocyte binding antigen-175 Region III-V	EBA-175 RIII-V	e175	Merozoite (Micronemes)	GST	3D7	(29)
Erythrocyte binding antigen-181 Region III-V	EBA-181 RIII-V	e181	Merozoite (Micronemes)	GST	3D7	(29)
Apical membrane antigen 1	PfAMA1	ama1	Merozoite (Micronemes)	His	FVO	(30)
Reticulocyte binding protein homologue 2	Rh2_2030	rh2030	Merozoite (Rhoptry)	GST	3D7	(31)
Reticulocyte binding protein homologue 4	Rh4.2	rh42	Merozoite (Rhoptry)	GST	W2mef	(32)
Reticulocyte binding protein homologue 5	Rh5.1	rh5	Merozoite (Rhoptry)	C-tag	3D7	(33)

*MSP, merozoite surface protein; kDa, kilodalton; GST, Glutathione S-transferase; iRBC, infected red blood cell; PVM, parasitophorous vacuole membrane*.

### Antimalarial Antibody Detection

Immunoglobulin G (IgG) data were collected for all participants as previously described (34). Antibody levels were measured using a MBA with a OneStep protocol enabling a rapid turnaround by incubating sample and secondary antibody simultaneously overnight (34). Median fluorescence intensity (MFI) was recorded using the MAGPIX with Bio-Plex Manager™ MP software (BioRad) and corrected for background reactivity to give a final value of MFI minus background (hereafter: MFI). Alongside participant samples, a 6-point serial dilution of a hyperimmune positive control pool (i.e., standard curve) was added to each plate to assess plate-to-plate variation in data collection.

### Quality Control of Antimalarial Antibody Data Collection

Plates with results that fell outside of the mean +/- 2 standard deviations (SD) of the third point of the serial dilution for two out of three highly immunogenic antigens (GLURP-R2, AMA-1 and MSP-1_19_) were repeated (15). Of the twenty-three *P. falciparum* antigens and peptides included in the panel, six had to be excluded as they showed loss in reactivity over time (previously described in van den Hoogen et al. (15) and for the remaining six antigenic targets shown in Supplementary Figure 1): MSP2_Dd2, MSP2_CH150/9, GEXP18, EBA-140 RIII-V, Rh2_2030, and Rh4.2. The remaining seventeen antigenic targets were analyzed for their association with cumulative and current or recent exposure to *falciparum* malaria.

### Statistical Analyses

#### Data Standardization

All statistical analyses were performed in R version 3.4.1 (35). After correction for background reactivity, the lowest MFI value recorded was−61, thus 65 was added to all values (i.e., for all antigens across all participants) and resulting MFI values were log_10_-transformed. Participants with high IgG responses to glutathione S-transferase (GST) were removed from further analyses (MFI>1,000) (15). In addition, results from participants below the age of 1 were excluded from the dataset to remove any influence of maternally derived antibodies. Finite mixture models were used to identify two components in the log_10_(MFI) participant data and the lower distribution was assumed to consist of seronegative individuals. Thresholds for seropositivity were defined as the mean of the lower distribution plus 5 SD (for details see Supplementary Methods in Supplementary Data Sheet 2).

#### Antibody Responses Associated With Cumulative Exposure

Antibody responses (log_10_MFIs) from participants were correlated with age; those that were most strongly correlated with age were considered to represent cumulative exposure. Analysis was conducted using the continuous antibody data instead of seropositivity to maximize the strength of any correlation. The HH-Artibonite survey was considered most informative in assessing correlations between antibodies and age, as it represents the full age-structure of the population sampled due to its community-based sampling strategy compared to sentinel group sampling in the EAG-surveys (children attending school and healthcare-seeking populations sampled at local facilities). Participants who were hsRDT positive were excluded to remove any antibody responses due to a current or recent infection. Spearman correlation coefficients (r) between all antibody responses and age (in years) were plotted using the R package *corrplot* (36). Only pairwise complete observations were used. The Bonferroni correction for multiple-comparisons was used to adjust the p-value for statistical significance. The chi-squared test for trend in proportions was used to test age-specific trends in seroprevalence [*stats* package in R (35)].

#### Antibody Responses Associated With Current or Recent Exposure

In absence of a gold standard for recent exposure (i.e., confirmed recent infection), hsRDT was used to determine antibody responses associated with current or recent exposure. The hsRDT detects histidine-rich protein 2 (HRP2), a *P. falciparum* antigen. HRP2 has been shown to persist in the blood up to weeks after parasite clearance following effective antimalarial treatment (37). The hsRDT has a lower limit of detection for HRP2 antigenemia (40–125 pg/mL HRP2) compared to the cRDT (600–1000 pg/mL HRP2) (38) and is thus potentially more sensitive to (chronic) low-density infections producing only low levels of HRP2. A positive hsRDT result may therefore indicate a current or recent infection. Antibody responses to antigenic targets that were not selected as cumulative exposure markers, were used to predict hsRDT positivity using receiver operating characteristic (ROC) curves. To assess utility to predict hsRDT infection status, only participant data from EAG-Grand'Anse was used, which was the survey with the highest rate of hsRDT positives. The EAG-Grand'Anse data was split into a training dataset (two-third, *n* = 3,201) and a test dataset (one-third, *n* = 1,648) using random selection. The numbers of hsRDT positive samples in the training dataset was 227 (7.1%) and in the test dataset was 120 (7.3%). The area-under-the-curve (AUC) of ROCs [*pROC* package; (39)] was measured using the training dataset in all ages as well as separately for those aged 1 to 15 years and those aged older than 15 years to assess any differences by age. The performance of the most informative antibody responses in predicting hsRDT status (i.e., AUC >0.8) was confirmed by repeating ROCs for the test, HH-Artibonite, and EAG-Artibonite datasets. Finally, ROCs using seropositivity endpoints were also assessed in the test dataset. ROCs were compared using DeLong's test for two correlated ROC curves [*roc.test* within the *pROC* package; (39)].

## Results

### Selection of Antibodies Associated With Cumulative Malaria Exposure

**Figure 1:**
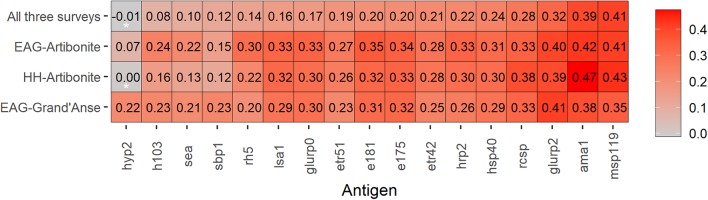
Heat map of Spearman correlation coefficients for age and immunoglobulin G (IgG) responses to seventeen *Plasmodium falciparum* antigens across three malaria transmission surveys in Haiti. IgG responses were defined as log10-transformed median fluorescence intensities corrected for background reactivity, while age is in years. Data is shown for 28,888 participants; highly sensitive rapid diagnostic test (hsRDT) positives were excluded (*n* = 536) to remove any IgG responses due to a current or recent infection. Coloring represents the strength of Spearman correlation coefficients from weak in grey to strong in red. Antigens (x-axis) are ordered from the lowest to highest Spearman correlation coefficient using data from all three surveys. Spearman correlation coefficients that were not statistically significant (i.e., *p* > 0.003 assuming Bonferroni correction for multiple testing) are indicated with an asterisk (^*^); all other age and IgG comparisons had *p*-values < 0.001.

In the HH-Artibonite survey, antibody levels, log_10_(MFI)'s, to most antigens showed weak positive associations with increasing age (Spearman correlation coefficient, *r* ≤ 0.40; Figure 1). AMA-1 and MSP-1_19_ showed moderate correlation with age (0.47 and 0.43, *p* < 0.001). The correlation coefficient between AMA-1 and MSP-1_19_ was 0.67, *p* < 0.001 (Supplementary Figure 2). Together with GLURP-R2, these antigens were consistently the top three in each survey, although MSP-1_19_ showed a weaker correlation (0.35, *p* < 0.001) than the other two antigens (≥0.38, *p* < 0.001) in the EAG-Grand'Anse survey. The validation of AMA-1 and MSP-1_19_ as the most appropriate markers of cumulative exposure is consistent with the literature across multiple settings (2, 40–42). A strong age-related increase in seroprevalence to the combined metric of cumulative exposure (i.e., seropositivity to either AMA-1 or MSP-1_19_) was seen across all surveys (chi-squared test for trend in proportions, *p* < 0.001).

### Selection of Antibodies Associated With Current or Recent Malaria Exposure

**Figure 2:**
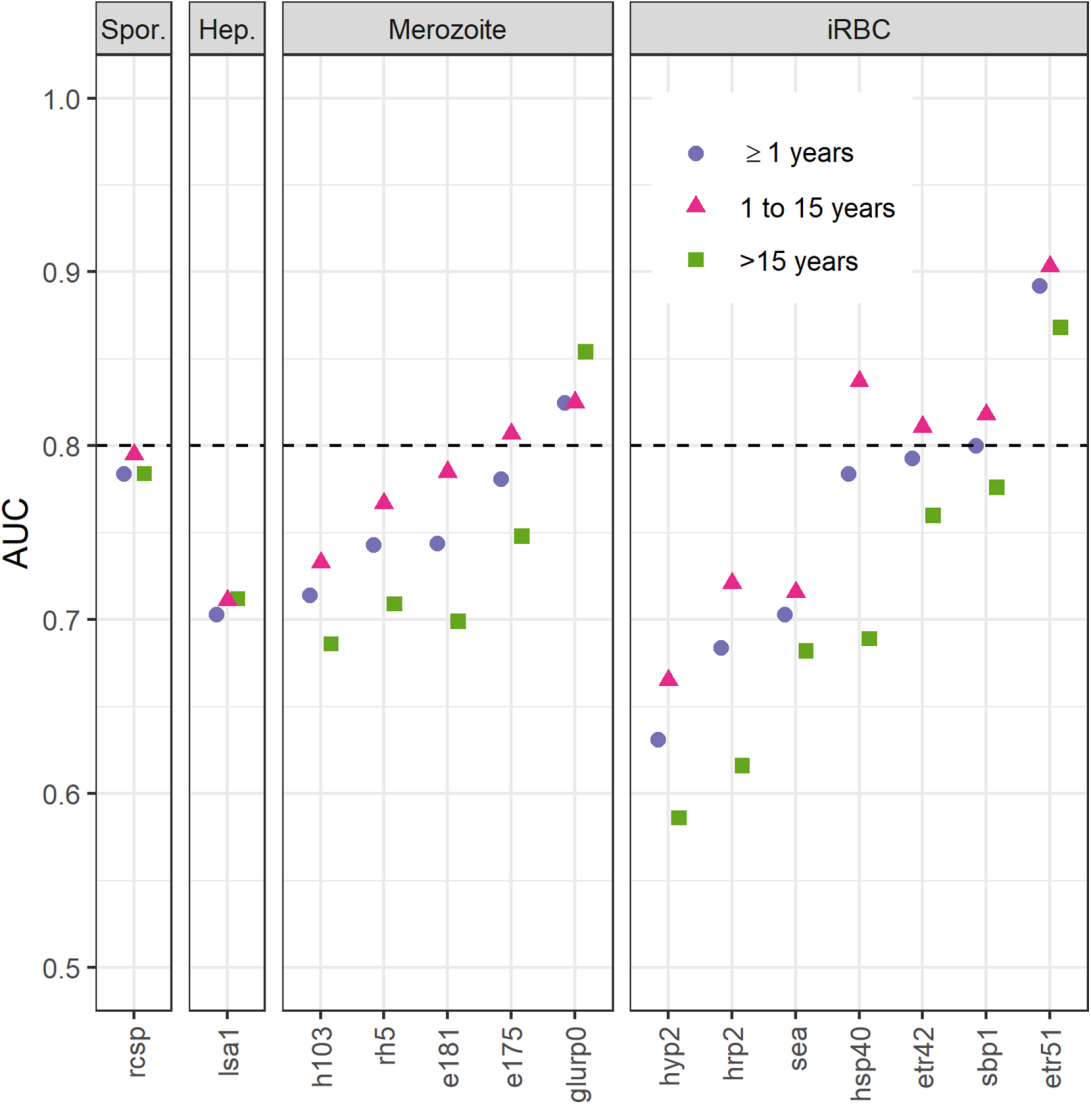
Area under the curve (AUC) of receiver operating characteristic curves for immunoglobulin G (IgG) responses to fourteen *Plasmodium falciparum* antigens using highly sensitive rapid diagnostic test (hsRDT) status as the gold standard. Antigens (x-axis) are ordered by parasite life cycle stage and AUC value for all ages. IgG responses (IgG) in two-thirds of all EAG-Grand'Anse participants were used (i.e., the training dataset; *n* = 3,201) including 227 hsRDT positives (7.1%). IgG responses were defined as log10-transformed median fluorescence intensities corrected for background reactivity. A threshold of 0.8 (dashed horizontal line) was used to select antigens for confirmation of results in the remaining one-third of the EAG-Grand'Anse dataset (see Figure 3). Spor.: sporozoite; Hep.: infected hepatocyte; iRBC: infected red blood cell.

Antibody levels to the remaining 14 targets were included to predict hsRDT status using ROCs and a training dataset consisting of two-thirds of the EAG-Grand'Anse participants. Etramp 5 ag 1 had the highest AUC for predicting hsRDT status (0.892), followed by GLURP-R0 (0.825); Figure 2. Responses to the remaining antigens had an AUC ≤ 0.8. For all antigens except GLURP-R0, the AUC for children aged 1 to 15 was greater than that for individuals aged 16 or older. There were clear differences in AUCs between these age groups for some antibodies (e.g., HRP-2 and HSP40) while for others AUCs were similar (e.g., LSA-1 and rCSP). For children aged 15 or younger—in addition to Etramp 5 ag 1 and GLURP-R0—SBP-1, Etramp 4 ag 2, HSP40 ag 1 and EBA-175 RIII-V had an AUC > 0.8.

**Figure 3:**
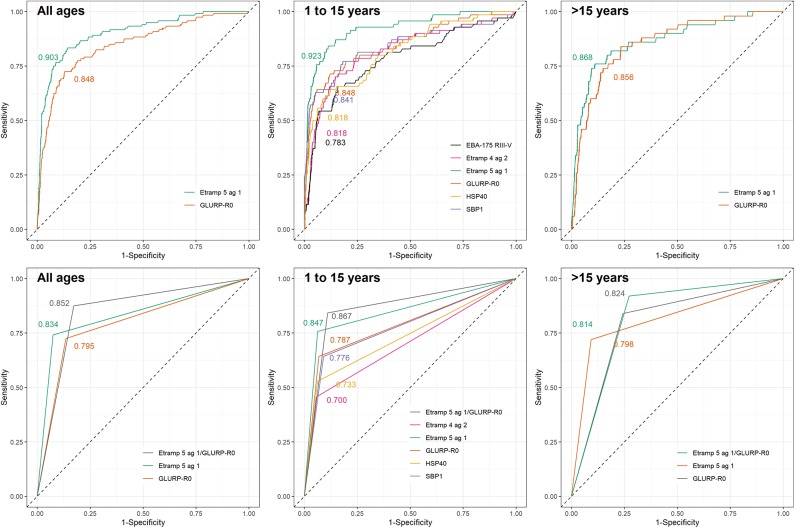
Receiver operating characteristic curves for immunoglobulin G (IgG) responses to selected *Plasmodium falciparum* antigens using highly sensitive rapid diagnostic test (hsRDT) status as the gold standard. IgG responses in one-thirds of all EAG-Grand'Anse participants were used (i.e., the test dataset; *n* = 1,648) including 120 hsRDT positives (7.3%). IgG responses were defined as log10-transformed median fluorescence intensities corrected for background reactivity. Results are shown for continuous IgG responses (top) for antigens with an area-under-the-curve (AUC) > 0.8 in the training dataset (Figure 2). Seropositivity results (bottom) are only shown for antigens with an AUC > 0.8 using continuous IgG responses in the test dataset (top). AUC values are depicted on each plot. From left to right results are shown for: all ages, children aged 1 to 15 years and individuals older than 15 years.

**Figure 4:**
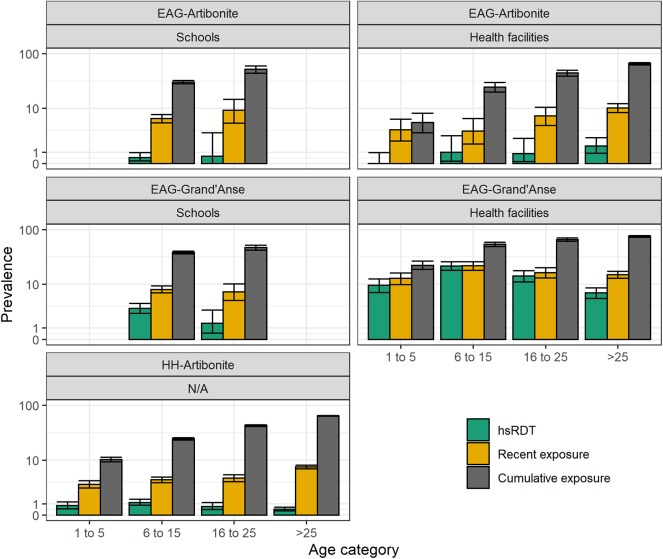
Highly sensitive rapid diagnostic test (hsRDT) prevalence as well as recent and cumulative exposure seroprevalence per age category (in years) and survey in Haiti. Recent exposure represents seropositivity to early transcribed membrane protein 5 (Etramp 5 ag 1) while cumulative exposure represents seropositivity to apical membrane antigen 1 (AMA-1) and/or the 19 kDa fragment of merozoite surface protein 1 (MSP-1_19_).

The performance of antibodies to Etramp 5 ag 1 and GLURP-R0 as accurate markers for hsRDT status (i.e., AUC>0.8) was confirmed in the test dataset (Figure 3) as well as the HH- and EAG-Artibonite surveys (Supplementary Figure 3). Using seropositivity, for all age categories, Etramp 5 ag 1 showed similar performance compared to GLURP-R0 (all ages: *p* = 0.118; children aged 1 to 15: *p* = 0.060; individuals aged 16 or older: *p* = 0.686) and the combined metric, representing seropositivity to either Etramp 5 ag 1 or GLURP-R0 (*p* = 0.266; *p* = 0.257; *p* = 0.733). As no improvement was seen by adding GLURP-R0, Etramp 5 ag 1 alone was selected to represent recent exposure to *falciparum* malaria. Age-specific hsRDT and seroprevalence per survey is shown in Figure 4.

## Discussion

This study used multiplex serological data to select antigen-specific antibody responses associated with cumulative and current or recent malaria exposure in Haiti. IgG responses from almost 30,000 participants were analyzed from three surveys across two locations with differing levels of malaria transmission intensity. Results showed that IgG responses to antigens AMA-1 and MSP-1_19_ best reflected cumulative exposure over time, whilst those to Etramp 5 ag 1 showed the highest accuracy in predicting current or recent infection when compared to hsRDT status. For the latter, survey data was used from the area experiencing the highest malaria transmission (Grand'Anse).

Continuous antibody responses to AMA-1 and MSP-1_19_ were confirmed as the optimum representation of cumulative malaria exposure as they showed the strongest correlation with age. These antigens have been used extensively in sero-surveillance for *P. falciparum* transmission (2). Seroconversion rates to AMA-1 and/or MSP-1_19_ have shown a strong correlation with household altitude (42), EIR (2, 42), child mortality (43, 44), the effect of malaria control interventions (43, 45), and patterns in malaria transmission over time (44). In addition, GLURP-R2 was moderately correlated with age. GLURP-R2 has been associated with protection from (symptomatic) malaria (46, 47) as well as cumulative exposure (40, 48). This study shows strong indications that seropositivity to AMA-1, MSP-1_19_, and to a lesser extent, GLURP-R2, represent cumulative exposure in this Haitian population. Some previous studies have found that AMA-1 and MSP-1_19_ can be good markers of active and recent infection in children (7, 8, 41), though at higher transmission, even in children, repeated exposure and subsequent antibody boosting limits their use as recent exposure markers (9). Given that we have confirmed the appropriateness of AMA-1 and MSP-1_19_ as a cumulative marker of exposure in Haiti, there is an added benefit of enabling comparisons of results across malaria-endemic settings due to their extensive use in sero-surveillance to date (2, 40–42).

Next, continuous antibody responses were used to validate markers of current or recent *P. falciparum* exposure according to hsRDT results. Etramp 5 ag 1 and GLURP-R0 were found to be most accurate in predicting hsRDT status in the EAG-Grand'Anse survey. As no further improvement was seen by combining seropositivity to GLURP-R0 and Etramp 5 ag 1, seropositivity to Etramp 5 ag 1 alone was selected as representative of current or recent exposure to malaria. For children – in addition to Etramp 5 ag 1 and GLURP-R0 – SBP-1, Etramp 4 ag 2, HSP40 ag 1, and EBA-175 RIII-V showed high accuracy in predicting hsRDT status. Intuitively, antibody responses in young children are more likely to represent a recent infection as they are unlikely to have developed a robust memory response following multiple infections (49), especially in areas of low transmission. Here we showed that all antibodies tested, except those to GLURP-R0, had higher accuracy in predicting hsRDT status in children compared to individuals aged 16 or older. Ideally, an IgG antibody response representing recent exposure does so across all ages as it minimizes the antigen panel needed. IgM antibody responses have regularly been suggested as a measure of recent exposure following their rapid acquisition and decline in viral infections, however, recent data showed IgM responses to malaria persisted over time and were not more short-lived than IgG responses to merozoite antigens (50).

Age-specific patterns of recent and cumulative exposure were consistent with expected exposure histories in the two transmission settings in Haiti: (1) recent exposure was higher in EAG-Grand'Anse compared to EAG-Artibonite which corresponds with the 2017 malaria incidence estimated at 18.1 and 0.6 per 1,000 inhabitants in Grand'Anse and Artibonite, respectively (source: National Malaria Control Program, PNCM, Haiti); (2) recent exposure was higher in healthcare-seeking populations compared to (likely asymptomatic) children attending school in Grand'Anse; (3) recent exposure was similar in children visiting a health facility or attending school in Artibonite, indicating that in this low transmission setting, healthcare-seeking is likely not driven by malaria disease; (4) cumulative and recent exposure was lowest in the HH-Artibonite survey representing an asymptomatic, community-based population across all ages.

The gold standard for identification of antibodies with short (or longer) half-lives, is assessment of antibody acquisition and decay rates in longitudinal studies following naturally acquired malaria infections. However, such studies are costly, time and labor intensive, and thus rare. Moreover, no existing studies have used the same combination of the age range of the study population, antigen panel assessed and assay conditions (9, 25, 40). Here we used hsRDT data from cross-sectional surveys to inform the selection of current or recent exposure markers. It was assumed that the lack in specificity in using antibody levels to predict hsRDT status represented recent malaria infections that had become hsRDT negative. Alternatively, these could be malaria infections below the limit of detection of the hsRDT. The lack in sensitivity might represent infections that are too recently acquired for an antibody response to have developed. Alternatively, certain individuals may not produce detectable antibodies to a specific antigen following infection. The overlap in identified recent exposure markers with previous longitudinal studies [Etramp 5 in Helb et al. (9) and GLURP-R0 in Kerkhof et al. (25)] is promising for selection and standardization of antigen panels for sero-surveillance (51). However, Kerkhof et al. also identified GLURP-R2 and MSP-1_19_ as recent exposure markers (25)—but these were better markers of cumulative exposure in our and other (42–45, 48) studies—indicating the need for caution and validation of these targets across populations and differing (histories of) transmission intensities. In settings with lower transmission, such as in Cambodia in the Kerkhof study (25), antibody levels are more likely to be reflective of recent exposure as they are not boosted following the lack of repeated exposure. This was also seen in our study, as nearly all antigen-specific antibodies analyzed accurately predicted hsRDT status in the Artibonite surveys where hsRDT prevalence was <1%.

There are different statistical techniques available to analyze multiplex antibody data. Boosted regression tree analysis has been used for *P. vivax* and *P. knowlesi* (52, 53). These studies showed that combined immune responses are more likely to be reflective of (recent) exposure though increments are small. It should be noted that the selection of antibodies for recent and cumulative exposure as described in this study is not meant to be exclusive. Remaining antibody responses may still be of interest for research-based studies interested in responses to, and patterns in, antibodies to specific targets. The antigens identified here were selected to enable rapid turnaround of analyses to directly inform malaria control programs in Haiti.

Here we described the selection of antibodies associated with cumulative and recent malaria exposure in Haiti. We used antibody responses (IgG) to 23 *P. falciparum* recombinant proteins and peptides from 29,481 participants across three surveys. In the absence of a gold standard (i.e., longitudinal data), we used age and hsRDT status to make this selection. Seropositivity to AMA-1 and/or MSP-1_19_ was selected to represent cumulative malaria exposure, while seropositivity to Etramp 5 ag 1 was selected to represent recent exposure to malaria. The identification of these antimalarial antibodies will simplify analyses and interpretation where results are needed to inform program decisions in Haiti.

## Data Availability Statement

The datasets generated for this study are available on request to the corresponding author.

## Ethics Statement

The EAG-Artibonite and EAG-Grand'Anse surveys were approved by the London School of Hygiene and Tropical Medicine (LSHTM) Research Ethics Committee (10393), Tulane Institutional Review Board (794709) and the National Bioethics Committee in Haiti (1516-30). The Center for Global Health Associate Director of Science reviewed and approved the protocols; Centers for Disease Control and Prevention (CDC) investigators were not considered to be engaged in human subjects research (protocol 2016-135a). The HH-Artibonite survey was approved by the CDC Institutional Review Board (6821), LSHTM Research Ethics Committee (10466) and the National Bioethics Committee in Haiti (1516-29 and 1617-31).

For the EAG-Artibonite and EAG-Grand'Anse surveys, consent was collected from school directors following community meetings to inform parents and allow opt-out. At health facilities, adult participants provided consent directly, and consent for children (<18 years) was provided by a parent or guardian and children above 6 years gave assent to participate. Individuals aged 16 or 17 who were married, head of household, or a parent were considered ‘mature minors’ and consented directly. Thumbprint consent or assent (countersigned by a witness) was used for illiterate participants. For the HH-Artibonite survey, verbal consent for overall permission to conduct the survey was obtained from the head of household and/or primary caretaker and documented in the electronic data collection instrument. Individual-level verbal consent was sought from each present individual for the blood sample collection, and assent from persons aged 7–17 years.

## Author Contributions

JL, MC, TPE, TD, RA, GS, CD, KH, and ER designed the surveys. TPE, TD, RA, VJ, GS, KH, and MC performed and supported field data collection. KT, JGB, SS and ER provided antigen constructs. JP, IR, TE, and GM performed laboratory data collection. LH, AE, JB, KT, CD, and ER supported laboratory data collection. LH, GS, KT, ER, and CD analyzed and interpreted the data. LH wrote the first draft of the manuscript with support from GS and CD. All authors read and approved the final manuscript.

## Conflict of Interest

The authors declare that the research was conducted in the absence of any commercial or financial relationships that could be construed as a potential conflict of interest.
