# Supplementary material for: Selection of Antibody Responses Associated With Plasmodium falciparum Infections in the Context of Malaria Elimination
Source: Front Immunol. 2020 May 15;11:928. doi: 10.3389/fimmu.2020.00928 (PMC7243477; doi:10.3389/fimmu.2020.00928)
Supplement: Supplementary file 1 [file Data_Sheet_1.docx]

**Supplementary Figures & Tables**

**Supplementary Table 1: Antigen to bead covalent coupling conditions for six antigens.** Conditions for the remaining antigens in the panel were previously reported in van den Hoogen, et al. [16].

| **Antigen** | **Alias** | **Coupling concentration (µg/ml beads)** | **Coupling pH** |
| --- | --- | --- | --- |
| EBA-140 RIII-V | e140 | 100 | 7.2 |
| EBA-175 RIII-V | e175 | 200 | 7.2 |
| EBA-181 RIII-V | e181 | 100 | 7.2 |
| Rh2_2030 | rh2030 | 50 | 5 |
| Rh4.2 | rh42 | 20 | 5 |
| Rh5.1 | rh5 | 10 | 5 |

**
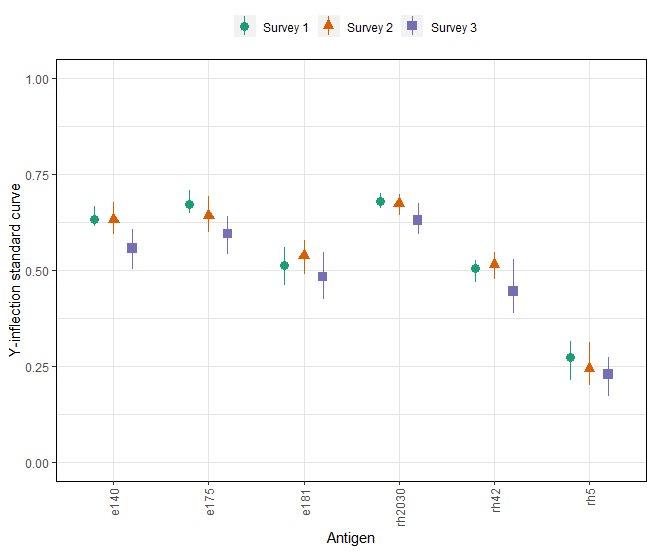
**

**Supplementary Figure 1: Median and interquartile range of predicted y-inflection points of standard curves per survey using a standard of Haitian hyperimmune sera.** A 6-point serial dilution of a hyperimmune positive control pool (i.e., standard curve) was added to each plate. For each plate and antigen, standard curves were fitted using 5-parameter logistic regression as described previously [16]. Median fluorescence intensity (MFI) values were converted to proportions using the minimum and maximum MFI value for all standard curves across all antigens (2.07 and 11.17 respectively). Standard curves were only fitted if the non-log-transformed MFI of at least one of the dilution points was larger than 100. Using the curve parameters, MFI values were predicted across a sequence of 200 values of standard curve concentrations for each of the plates. Median (shapes) and interquartile range (vertical lines) of the predicted y-inflection points from standard curves across all plates are shown per survey. An antigen failed quality control if the median of the y-inflection point of the third survey was lower than the 25^th^ percentile of the other two surveys. For antigen (x-axis) acronyms see Table 2.

**
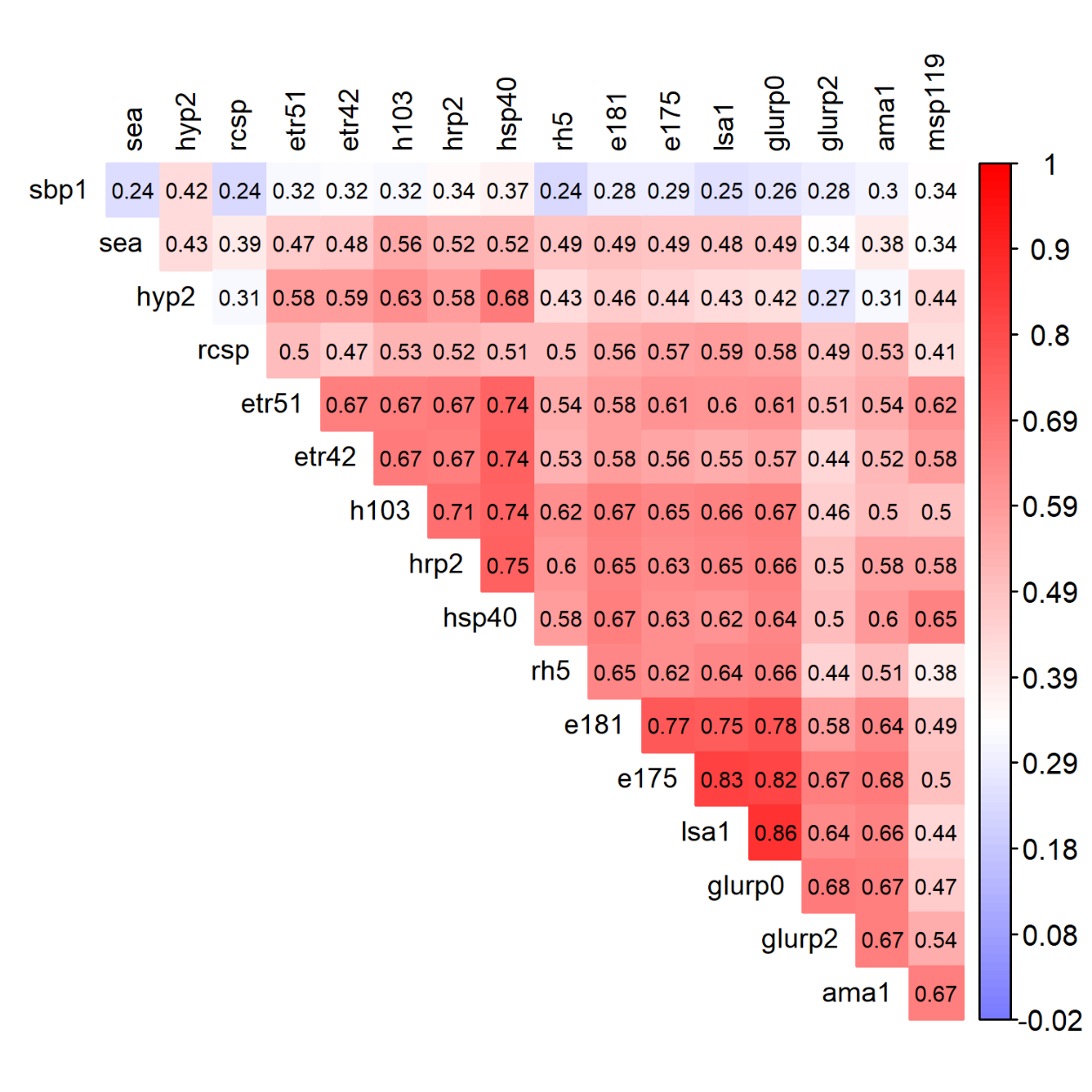
**

**Supplementary Figure 2: Heat map of Spearman correlation coefficients for the association between IgG responses to seventeen *Plasmodium falciparum* antigens using data from three malaria transmission surveys in Haiti.** Antibody responses (IgG) were defined as log10-transformed median fluorescence intensities corrected for background reactivity. Data is shown for 28,888 participants; highly sensitive rapid diagnostic test positives (hsRDT) were excluded (n=536) to remove any antibody responses due to a current or recent infection. Colouring represents the strength of Spearman correlation coefficients from weak in blue to strong in red.

**
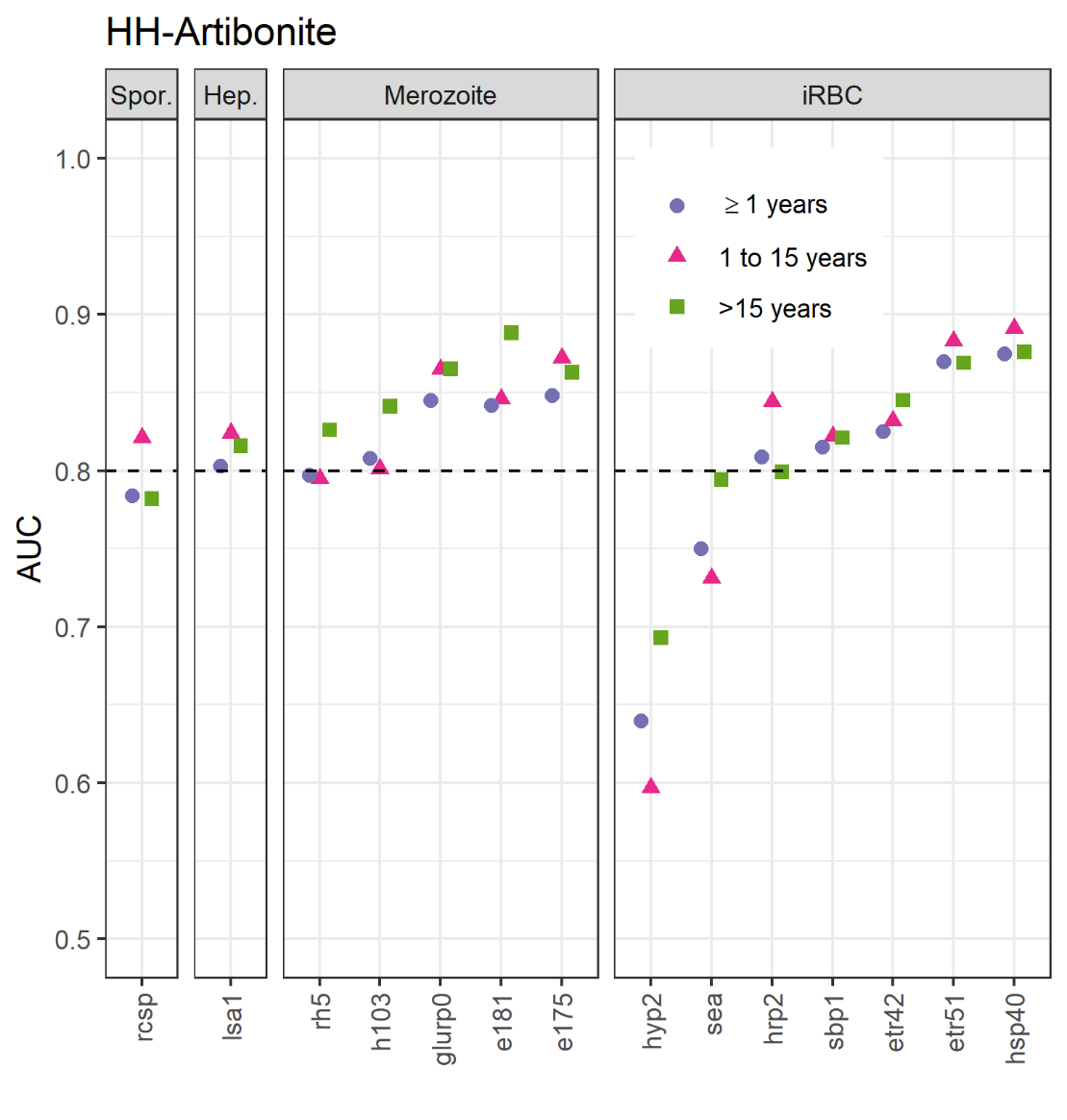
**

**Supplementary Figure 3: Area under the curve (AUC) of receiver operating characteristic curves for IgG to fourteen *Plasmodium falciparum* antigens using highly sensitive rapid diagnostic test (hsRDT) status as the gold standard in the Artibonite surveys.** Antigens (x-axis) are ordered by parasite life cycle stage and AUC value for all ages. Antibody responses (IgG) from HH-Artibonite (n=20,609) and EAG-Artibonite (n=4,019; next page) participants were used and were defined as log10-transformed median fluorescence intensities corrected for background reactivity. A threshold of 0.8 (dashed horizontal line) was used to define informativeness of an antigen in predicting hsRDT status. Spor.: sporozoite; Hep.: infected hepatocyte; iRBC: infected red blood cell.

**
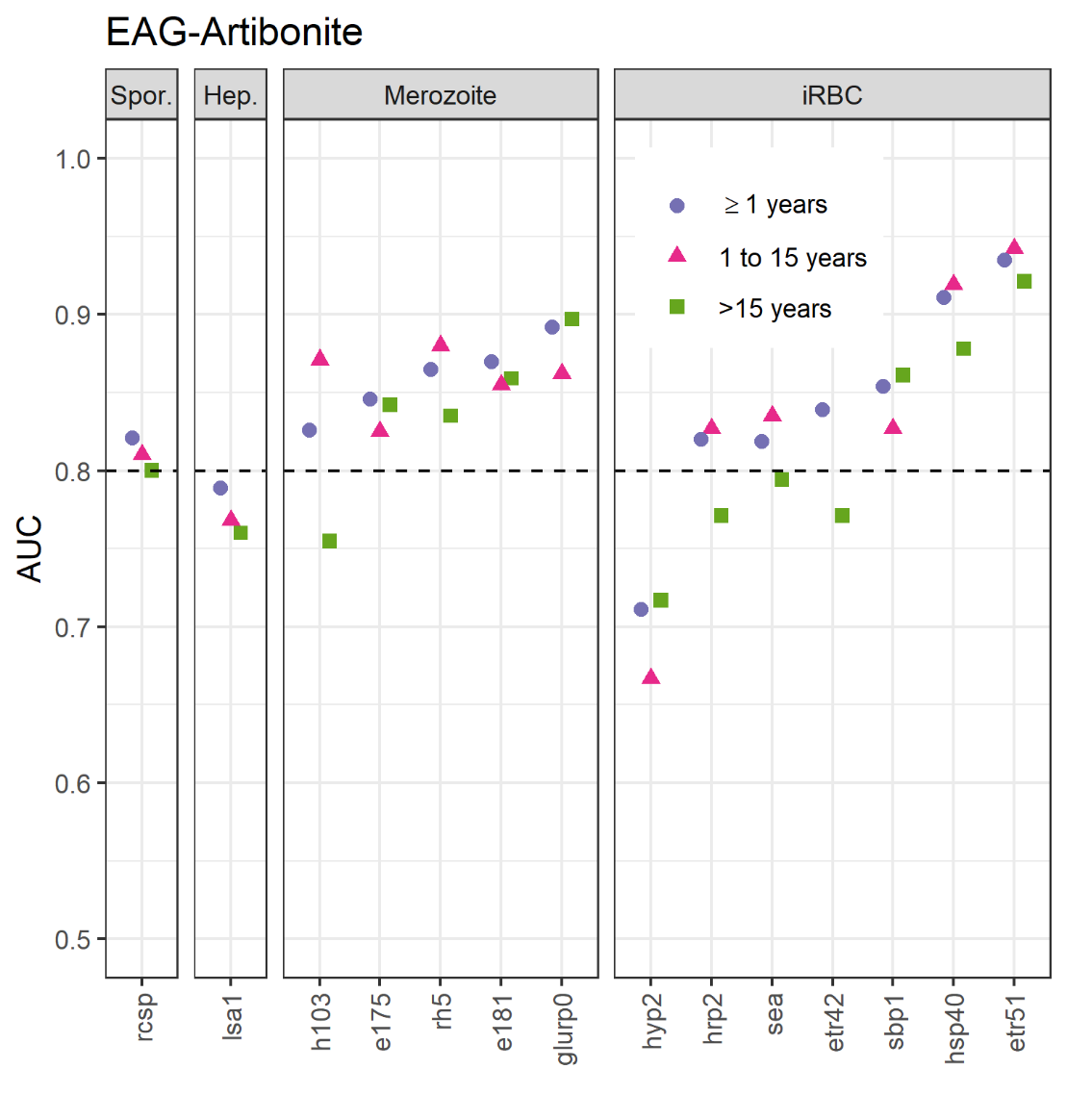
**

**Supplementary Figure 3** (continued).
