# Supplementary material for: Selection of Antibody Responses Associated With Plasmodium falciparum Infections in the Context of Malaria Elimination
Source: Front Immunol. 2020 May 15;11:928. doi: 10.3389/fimmu.2020.00928 (PMC7243477; doi:10.3389/fimmu.2020.00928)
Supplement: Supplementary file 2 [file Data_Sheet_2.docx]

**Supplementary Methods: Defining seropositivity across surveys performed in differing malaria transmission levels**

Median fluorescence intensity (MFI) measurements were corrected for background reactivity and log10-transformed (MFI-bkg). Density distribution plots showed that the lower Gaussian distribution of the MFI-bkg data from each of the three surveys were not aligned (example for MSP-1_19_ shown in Figure 1; top). In order to compare surveys directly, antibody distributions were aligned according to the mean of the lower distribution. This was done by adding a constant value to all MFI-bkg measurements per survey to adjust for the difference in the means of the lower distributions, using the EAG-Grand’Anse survey as the reference (Figure 1; bottom). Hereafter, a two-Gaussian distribution was fit to the combined MFI-bkg data (i.e., across all three surveys) and the threshold for seropositivity was set at five standard deviations from the mean of the lower Gaussian distribution (Figure 1; bottom).

This approach was applied to each of the antigens in the panel separately. Resulting thresholds for seropositivity are shown in Table 1.

**
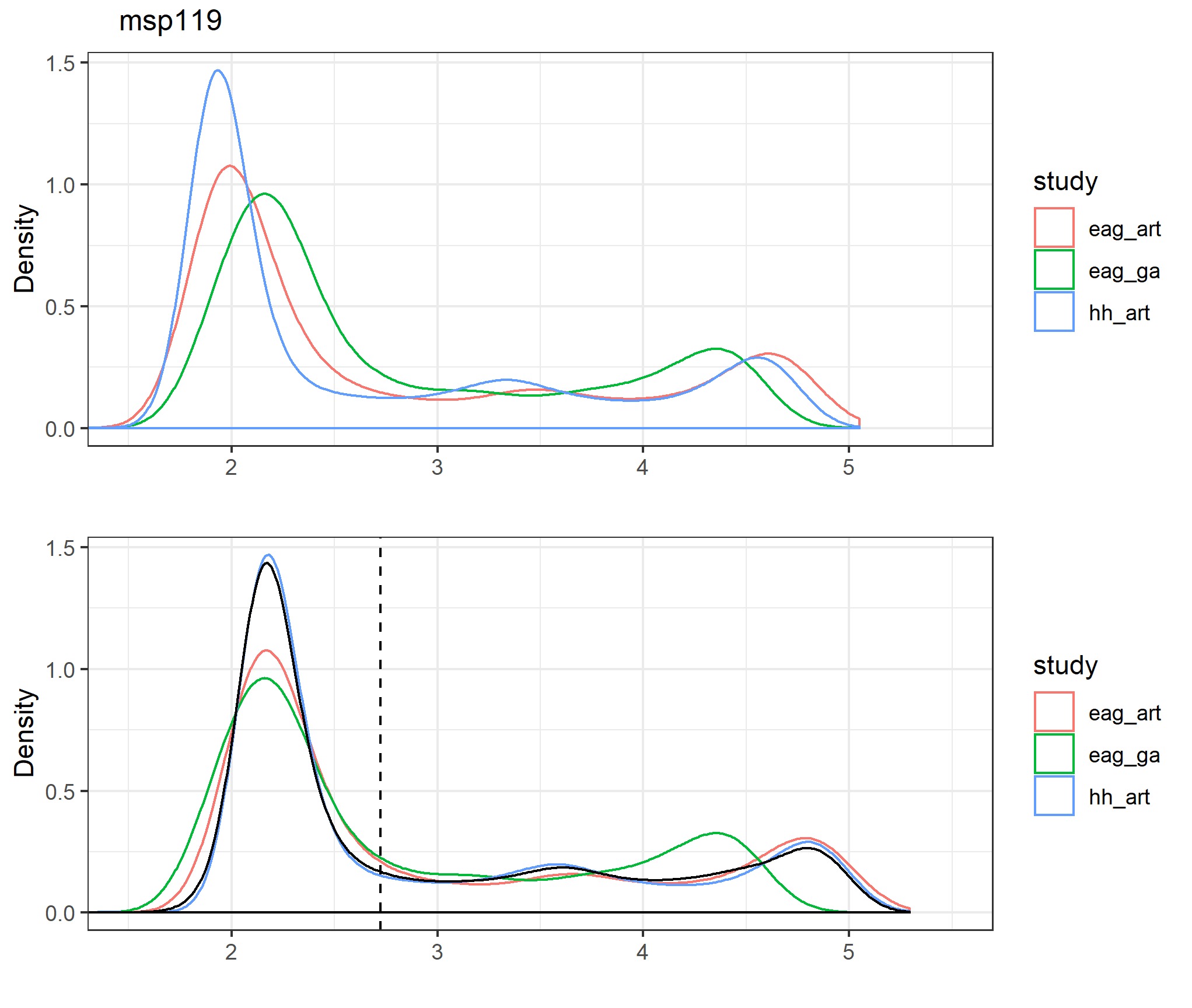
**

**Supplementary Methods Figure 1: MSP-1_19_ antibody density distributions per survey (top) and aligning antibody distributions resulting in a combined antibody density distribution across all three surveys (bottom; in black).** The threshold for seropositivity (dashed vertical line in bottom plot) was determined at five standard deviations from the mean of the lower Gaussian distribution using the combined antibody density distribution across all three surveys (black solid line). MSP-1_19_/msp119: 19 kDa fragment of Merozoite Surface Protein 1.

**Supplementary Methods Table 1: Overview of thresholds for seropositivity by applying a two-Gaussian mixture model per antigen using the combined IgG data across all three surveys.** Thresholds are presented in log10-transformed median fluorescence intensity (MFI).

| **Antigen** | **Antigen acronym** | **Alias** | **Seropositivity threshold** |
| --- | --- | --- | --- |
| Circumsporozoite surface protein | rCSP | rcsp | 2.654 |
| Liver surface antigen 1 | LSA-1 | lsa1 | 2.394 |
| Plasmodium exported protein | Hyp 2 | hyp2 | 2.697 |
| Heat shock protein 40 | HSP40 Ag1 | hsp40 | 2.606 |
| Schizont egress antigen | SEA-1 | sea | 2.758 |
| Skeleton-binding protein; Maurer's cleft | SBP1 | sbp1 | 2.815 |
| Histidine rich protein 2 | HRP2 | hrp2 | 2.739 |
| Early transcribed membrane antigen | Etramp 4 ag2 | etr42 | 2.883 |
| Early transcribed membrane antigen | Etramp 5 ag1 | etr51 | 2.566 |
| Glutamate rich protein R0 | PfGLURP R0 | glurp0 | 2.418 |
| Glutamate rich protein R2 | PfGLURP R2 | glurp2 | 2.236 |
| 19kDa fragment of MSP1 molecule | PfMSP1-_19_ | msp119 | 2.724 |
| H103/merozoite surface protein 11 | H103/MSP11 | h103 | 2.631 |
| Erythrocyte binding antigen-175 Region III-V | EBA-175 RIII-V | e175 | 2.279 |
| Erythrocyte binding antigen-181 Region III-V | EBA-181 RIII-V | e181 | 2.509 |
| Apical membrane antigen 1 | PfAMA1 | ama1 | 2.461 |
| Reticulocyte binding protein homologue 5 | Rh5.1 | rh5 | 2.792 |
